# Discussion on the boundary of risk assessment and risk management

**DOI:** 10.1186/s41021-015-0008-6

**Published:** 2015-07-30

**Authors:** Jun Kanno

**Affiliations:** Division of Cellular & Molecular Toxicology, Biological Safety Research Center, National Institute of Health Sciences, Kamiyoga 1-18-1, Setagaya-ku, Tokyo 158-8501 Japan

**Keywords:** Risk assessment, Risk management, Risk communication, Risk analysis, Toxicology

## Abstract

The current question on the relation of “risk assessment” and “risk management” may exist at the level of who should be responsible for what. Here, the argument will be made upon “Risk Analysis”. The problem here is whether a same person can or should perform both risk assessment and risk management or not. The risk assessors and risk managers are to be sharing the task to perform the risk analysis under close cooperation. The practical endpoint of the risk analysis is the “round table meeting” where the risk assessors and risk managers become aware of their own accurate role and relation with the other stake holders inclding those who are managed or regulated.

Credentials needed for a toxicologist to be a risk assessor are not only an ability to qualify and analyze the data, but an ability to understand the ‘plausibility’ that can be extrapolated from them. Different from the “weight of evidence” approach, plausibility is to extrapolate the data by scientifically referring to the wider range of knowledge.

Even as a perfect risk assessor, a tragedy waits for those who unknowingly mixes up the position of risk assessor and risk manager. Potentially it happens when a personal belief was reflected to risk management and went wrong with the risk communication processes. In the round table, risk assessors and managers should talk to each other with plausibility in science and sharing the task towards the goal of the meeting.

A cascade of events initiated by the March 11, 2011 devastating earthquake has affected toxicology researchers in various ways. A generalized conclusion here is that the risk assessors and risk managers should discuss, before and during the round table meeting, “how far can we pollute our living space?” based on a realization that Toxicology is covering both “Hygiene” of public population and “Clinical aspects” of individuals who consult a doctor.

## Discussion on the boundary of risk assessment and risk management

“… The Risk Assessment Report on Radioactive Nuclides in Foods, Food Safety Commission (FSC) of Japan, October 2011 is merely a data trove, and lacks the form of risk assessment document. It concluded that ‘more than around 100 mSv of extra cumulative effective dose could increase the risk of effect on health’ and that ‘the amount smaller than 100 mSv cannot be assessed’. It did not assess how much of excess risk will be seen above 100 mSv either (from the public comment to the Report)…”[1, 2]

There are various opinions about how “risk assessment” and “risk management” should be. However, the current problem may exist at the level of who should be responsible for what. Here, the argument will be made upon the proposal on “Risk Analysis” by the 1995 Report of the Joint FAO/WHO Expert Consultation “Application of Risk Analysis to Food Standards Issues” [3]; adopted by 2003 Japanese Food Safety Basic Act [4]. Risk analysis consists of three elements, risk assessment, risk management and risk communication. Risk assessment is to scientifically evaluate known or potential adverse health effects resulting from human exposure to a certain agent. The definition includes quantitative risk assessment, which emphasizes reliance on numerical expressions of risk, and also qualitative expressions of risk, as well as an indication of the attendant uncertainties. Risk assessment is performed on the basis of scientific data mostly derived from toxicological studies or tests on the agent of interest. Where there is uncertainties due to an insufficiency of available data, it is a task of risk assessors to set plausible assumptions to compensate for the data gaps. Therefore, toxicology is regarded as the driving force of the risk assessment and eventually of the risk analysis. Risk management is the process of weighing policy alternatives for the acceptance, minimization or reduction of the assessed risks and for the selection and implementation of appropriate options. Finally the risk communication is the interactive process for the exchange of information and opinion on the risk among risk assessors, risk managers, and other interested parties including those who are managed or regulated.

The problem according to the topic here is whether a same person can or should perform both risk assessment and risk management or not. Ideally speaking, risk assessment should be executed by scientific (toxicology) experts and risk management by persons who are neutral, impartial and capable of transparent discussion, and most of all, those who can be responsible for the outcome of the management acts. The risk assessors and risk managers are sharing the task to perform the risk analysis under close cooperation. Although initially, the FSC was told to perform risk assessment, it is now legally entitled to perform both assessment and management [4]. When a single expert scientist or researcher conducted both roles, i.e. risk assessor and risk manager at the same time, the person becomes liable to the act of management. This situation may affect the position of the expert thereafter; a “biased” or “labeled” person. In this context, a term “Regulatory Science” can be narrowly defined as “a science to establish methods for clear and close communication between the risk assessors and risk managers to avoid unnecessary and unsavory ties between them.”

The practical endpoint of the process of risk analysis is the “round table meeting” where it is vital for the risk assessors and risk managers to know the accurate role and relation with the other stake holders.

In case of risk assessment of drugs, for example, go-no-go decision in the process of drug development, the most important information for the risk assessors is the mechanistic data, such as the target of toxicity, not the data from regular guideline toxicity studies. In case of food itself (excluding food additives and other known artificial and natural contaminants) its risk assessment has been conducted using the knowledge of “eating experiences”. Such “food” has been consumed at an amount of grams or tens of grams per kg body weight. It is, therefore, impossible to expose experimental animals with the food at an amount 100 times more than the human consumption in order to obtain toxicity data to set the safety factor of 100. Some new functional food should be assessed based on the mechanistic data, just like pharmaceuticals, both on beneficial effects and toxicological effects. If any toxicity is suspected, any available methods, including high sensitivity transgenic mouse and transcriptomic analysis, often mechanism-specific in sensitivity, should be used to confirm the toxicity. A typical case was the diacylglycerol (DAG)-enriched edible oil. It was found to contain relatively high concentration of glycidol-fatty acid ester (GFA). Glycidol (G), an epoxide compound, was well-known for its carcinogenicity. At the beginning of the assessment process, the contamination of the oil by GFA was not known. Instead DAG itself was questioned for its possible cancer promotion activity via protein kinase C signaling pathways. Various high sensitivity test were conducted on the oil, and some of them showed positive results. Some toxicologists concluded that because it is only a few tests that were positive, it is likely that they are false positive. The other toxicologists argued that the positive results are indicating the mechanistic possibility that the oil is biologically active against certain signaling system which can be related to toxicity. Then came in the information of GFA. Although it was highly plausible that GFA is hydrolyzed to G and show mutagenicity, the FSC took some considerably long time to run tests to confirm the mutagenicity of GFA [5].

What are the credentials needed for a toxicologist to be a risk assessor? At least, an ability to read data and to judge whether the study was conducted properly. But it turned out that it is not enough. It seems essential to understand the limitation of a traditional toxicity study by reading its protocol. In other words, an ability to point out a need of additional mechanistic test endpoints for it. From a wider view, it can be rephrased as “an ability to understand the ‘plausibility’ that can be extrapolated from the available data”. The limitation of “weight of evidence” approach would lie within its definition that it is an approach to interpolate within available data from related toxicity studies. Plausibility, on the other hand, is to extrapolate the data by scientifically referring to the wider range of knowledge. In the case of GFA mutagenicity, there was no direct data on GFA, but sufficient scientific knowledge that GFA can be hydrolyzed by lipases to G and FA and hence show mutagenesis. Now the problem there should have been whether G was an element responsible for positive data of some mechanistic tests performed on the oil. Generally speaking, such detour in the process of risk assessment would be forced by a certain stake holder.

A tragedy awaits for a decent scientist who unknowingly mixes up the position of risk assessor and risk manager. It happens especially when a scientist’s honest belief was reflected to risk management but did not go well in the end at the level of risk communication with the public. Such scientist becomes difficult to go back to “a neutral position” from the public standpoint. To exacerbate such processes, mass media often starts to control information not only following suggestions by the managers but also, interestingly, by an opportunistic self-regulation.

The other side of the risk analysis is the public. The problem, sometimes, with the public is its “laziness”, especially to a less urgent safety issue; the public sometimes looks like they do not want to hear, learn, understand, judge, and take any responsibility. One outcome of such trend is the “Zero Risk”. Some risk assessor toxicologists see that the public prefers “Zero Risk” so that the assessment should match that demand. Consequently such assessors campaign to set threshold for any toxicity including carcinogenicity even in the scientific fields. However, are the public so lazy and easygoing for highly urgent issues? Apparently not. Even though, one cause of the failure of the round table meeting is the strong concerns that the act of joining itself would become an indication of some agreement with the manager. It is said that the key to organize a successful round table meeting is to set a common aim agreeable for all the participants/stakeholders. A practical aim can be limited to “for the public”, “for the country”, or “for the safety and peace of mind (security) of all citizens”. And also a proper chair person who can be neutral, non-biased and trusted. In the round table, risk assessors and risk managers should talk to each other with an understanding of the importance of plausibility in science sharing the task towards the goal of the meeting. It is often cited that the United Kingdom establishment of the Scientific Advisory Group for Emergencies after the crisis of BSE (bovine spongiform encephalopathy) issue is a possible prototype towards the round table meeting for handling emergencies [6, 7].

## Discussion

A lesson learned from the Fukushima incidence was that the “peace of mind” can be easily lost and becomes difficult to restore when the communications procedure was ignored by the assessors and/or by the managers. Under the “Safety Myth” of the nuclear power, linear non-threshold (LNT) hypothesis was told to the public for long time. A sudden imposition of threshold-like number of 100 mSv was instinctively rejected, or at least, welcomed by deep confusion, resulted in the public mistrust not only of the governmental measures but also of the actions made by scientists/experts. It became clearer than ever that the round table meeting should have been conducted properly with transparency, fairness, and clear positioning of the responsibility of any act decided by the meeting. Mass media should have been responsible to broadcast the process of the meeting.

## Conclusion

**Fig. 1:**
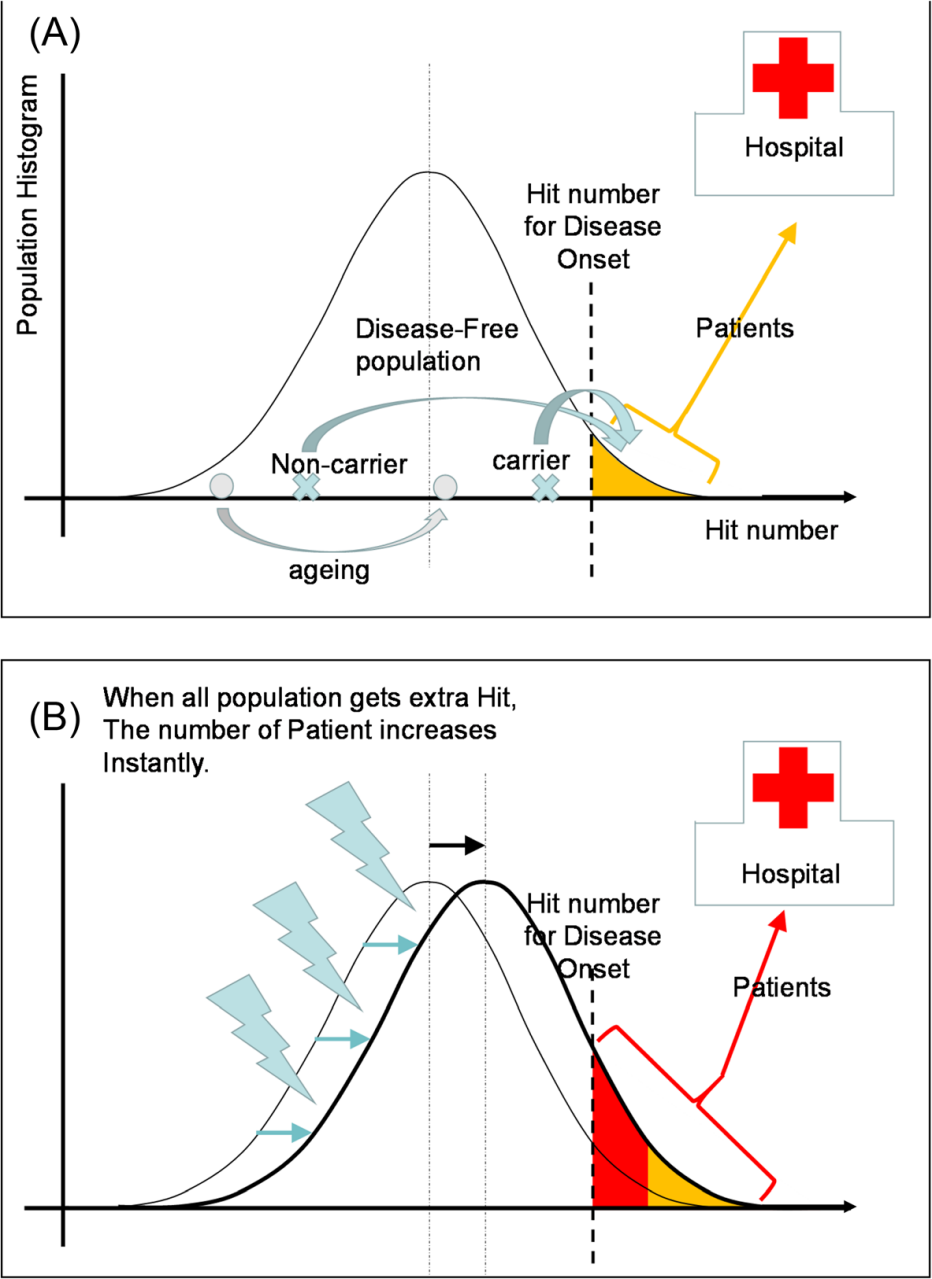
The toxicology should look over the entire population including both the “disease-free population”, a target area of “hygienic” sciences, and the “patients”, a target area of “clinical” studies, as one continuous multifactorial entity. (**a**) A schematic presentation of the population with various degrees of hits towards the onset of a certain disease. The shape of the distribution may vary according to the type of disease and the hit number required for the onset of the disease. Here, the normal distribution for a multifactorial disease is postulated. Within the disease free population, an individual can accumulate more hits by ageing or by environmental insults (grey dot). At birth, genetic condition can vary among individuals such as non-carrier and carrier. Carriers are closer to the border between disease-free population and patients who need medication. (**b**) When the entire population, i.e. all individuals in the normal distribution, gets extra hit by a certain agent, there is a sudden increase in the number individuals who cross the line of onset line and become ill

Speaking more generally, apart from the specific issues above, and as a conclusion here from the toxicological point of view, a question that the risk assessors and risk managers should discuss before and during the round table meeting is “how far can we pollute our living space?” As shown in Figure 1, all members of the public are accumulating hits towards developing a disease. It can be inherited genetic hits, chemicals, radiation, or hits that increases “merely by ageing”. There is a number of hits required to develop a certain disease, and relatively a small portion of the population are hospitalized for it. If all the population are exposed to a certain insult that adds one more hit, a small but clearly monitorable portion of population will newly develop the particular disease and have to go to a hospital. This increase in patient number can be monitored by the clinical doctors in the hospital with a very high sensitivity; it is said that a new disease entity is reported when three cases are accumulated. On the other hand, it is very difficult for the rest of the people who are apparently healthy to sense that a few of them became ill due to one additional hit and went to a hospital. The risk assessors and risk managers should be aware of both the new patients and the apparently healthy population who is steadily gaining hits towards disease development.
